# Intravascular, Interstitial and Intracellular Volume Changes During Short Term Deep Tissue Massage of the Calf: A Case Study

**DOI:** 10.2478/joeb-2022-0011

**Published:** 2022-11-23

**Authors:** Leslie D. Montgomery, Rowena Galang, Richard W. Montgomery

**Affiliations:** 1 LDM Associates, San Jose, CA, USA; 2 National Home Health Services, San Jose, CA, USA

**Keywords:** Deep tissue massage, compartment volumes, bioimpedance

## Abstract

The following case study demonstrates that the effectiveness of Deep Tissue Massage (DTM) can be monitored in real time with bioimpedance. DTM techniques are used as a medical treatment to help reduce swelling of the calves of congestive heart failure patients. Bioimpedance monitoring shows immediately how fluid is redistributed within the intravascular, interstitial and intracellular fluid compartments, and how long the redistribution lasts. Bioimpedance spectroscopy, as used in this study, is a non-invasive procedure that can be used to monitor compartment fluid volumes and changes during many fluid management procedures.

## Introduction

### Massage

There are numerous types of physical massage modalities. Most massage therapies are done to relieve stress, reduce inflammation, ease sore muscles and/or restore the body's energy. Only Deep Tissue Massage (DTM) and Deep Lymphatic Massage (DLM) are done to reduce the fluid accumulation that may take place in different segments of the body.

DTM differs from DLM in its purpose, focus and mode of application [1]. DTM employs a greater pressure applied around the leg in slow strokes from the ankle toward the heart. DLM uses a gentler "kneading" type force applied to the skin surface to return accumulated fluids back into the lymphatic drainage system [2].

Little information is available regarding the depth or effectiveness of either DTM or DLM. The objective of this pilot level study was to assess how well this information can be obtained using the bioimpedance device described below. In particular, how DTM affects the three fluid compartments that may accumulate fluids in congestive heart failure patients. Congestive heart failure not only leads to fluid retention but also triggers a change in fluid distribution (intra- and extra-cellular and vascular fluid shifts).

### Bioimpedance

An electrical impedance spectrograph (EIS) (Z-Scan-2, U.F.I. Inc, Morro Bay, CA) was used to monitor segmental intracellular and extracellular compartment volumes. A custom analytical program was then used to divide the extracellular compartment volume into its intravascular and interstitial components. Detailed descriptions of the EIS and analytical procedures used in this study can be found in Sasser and Gerth [3], Gerth and Watke [4] and Fricke [5,6]. The EIS was previously validated [7] and used [8,9] to monitor fluid shifts between the intracellular, interstitial, and intravascular compartments during dialysis.

## Methods

### Subject characteristics

The subject that took part in this pilot level study is a male who has congestive heart failure. He is 83 years old, weighs approximately 130 pounds and is 5 ft. 9 in. tall. He is on a limited fluid intake regime of 2 liters/day and closely monitors his food (sodium) intake to be under 600 – 800 mg/day. He is taking a diuretic in the form of 100 mg oral Torsemide (DEMADEX) administered orally once a day in the morning. This dosage is adjusted based upon his current weight, swelling and blood pressure. The goal for blood pressure is to maintain it at approximately 95/65 throughout the day.

**Figure 1:**
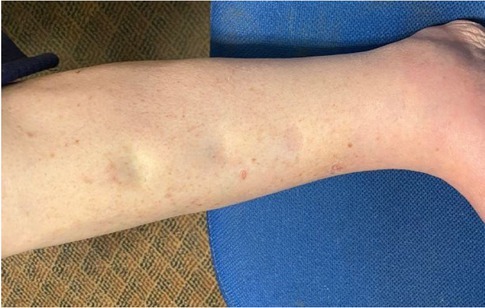
Extent of pitting edema in calf prior to DTM.

To date, this program has been rather successful in preventing severe edema in the thigh, torso and chest. However, by the end of the day his calves exhibit mild pitting edema of approximately 1 mm in depth (Figure 1).

### Massage technique used

The objective of DTM is for the pressure applied by the therapist to be more fully transmitted to the underlying muscles where it can mobilize and move the accumulated fluids back toward the heart [2].

**Figure 2:**
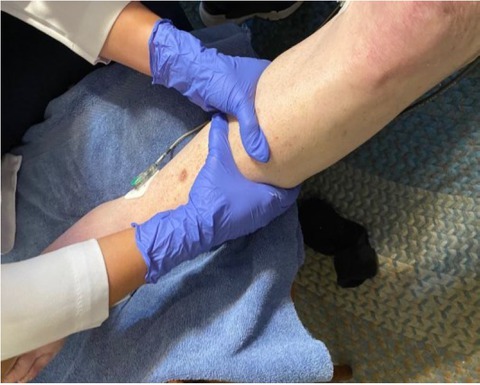
Position of hands during DTM.

The DTM in this study was administered as described by The Wellness Council [10] and Healthline [11] using the following steps:

1. The leg is placed in a horizontal position and allowed to rest prior initiation of the massage,
2. Aveeno (dimethicone skin protectant – Johnson & Johnson Co.) daily moisturizing lotion was then used to lubricate the calf before and during the deep tissue massage.
3. The therapist then applied circumferential force to the calf through long slow strokes from the ankle toward the knee using the hands and thumbs as shown in Figure 2.
4. DTM was continued for five minutes after which the leg remained horizontal for a recovery period.
5. After the recovery period the subject sat upright with the leg perpendicular to the floor
6. to allow the fluid in the calf to return to pretest levels.

The timing sequence of the various phases of the DTM was:

Elapsed Time (minutes):

**Table:** 

0	start treatment session - placed leg on chair
15	start massage and end of instrumentation period
20	stop massage - start recovery
60	placed leg on floor
120	end of treatment session

### Bioimpedance instrumentation

**Figure 3:**
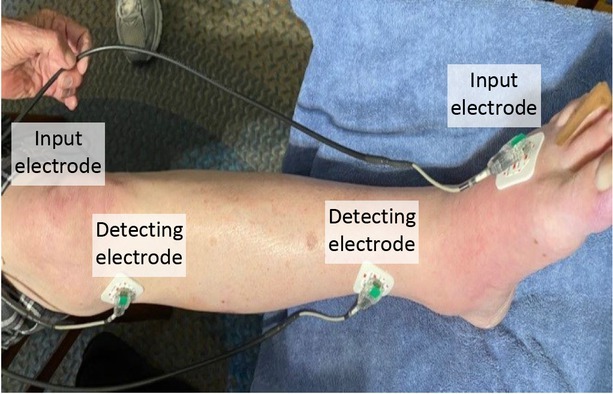
EIS electrode placement on the calf.

The EIS was applied to the calf by using four ECG electrodes attached to the subject’s dominant lower leg as shown in Figure 3. An extraneous current is passed between the two (input) electrodes placed just above the knee, and just above the lateral malleolus. The resistance and reactance of the calf was measured between the two (detecting) electrodes placed just below the knee and above the ankle [12].

### Informed consent

Informed consent has been obtained from all individuals included in this study.

### Ethical approval

The research related to human use has been complied with all relevant national regulations, institutional policies and in accordance with the tenets of the Helsinki Declaration.

## Results and discussion

To our knowledge, this is the first study to use bioimpedance to simultaneously quantify the intravascular, interstitial and intracellular fluid compartment volumes during DTM. DTM was found to affect each fluid compartment volume in two different ways: the amount of the fluid change during the five-minute application of DTM (Table 1) and the time period between initial DTM and its effect on the individual compartment volumes.

**Table 1:** Vblood, Vcell, Vinstl Volumes and Changes in Volume (all mL) at Elapsed Times (min.) of changes in the DTM treatment.

ELAPSE TIME - Min.	V_blood_	Change in V_blood_	V_cell_	Change in V_cell_	V_instl_	Change in V_instl_
0	1035	0	926	0	1067	0
15	998	-37	907	-19	1054	-13
20	1013	15	816	-91	994	-60
60	1006	-7	556	-260	1001	7
120	957	-49	702	146	1033	31

The salient findings of the current study are as follows:

## 1 The EIS system and procedures, as used in this case study, can be used to quantify the intravascular, interstitial and intracellular fluid compartment volumes during DTM.

**Figure 4:**
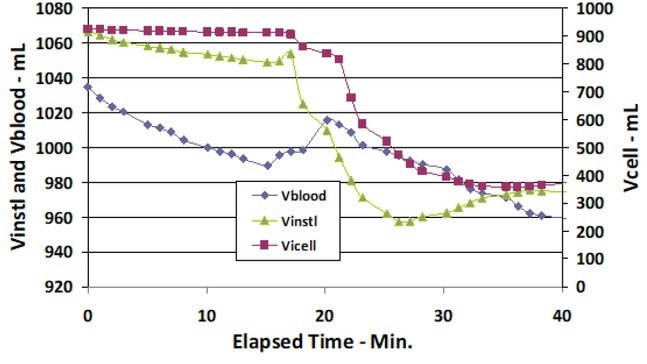
Vblood, Vinst) and Vcell volumes (mL) of the calf during start of DTM vs. Elapsed Time (min).

**Figure 5:**
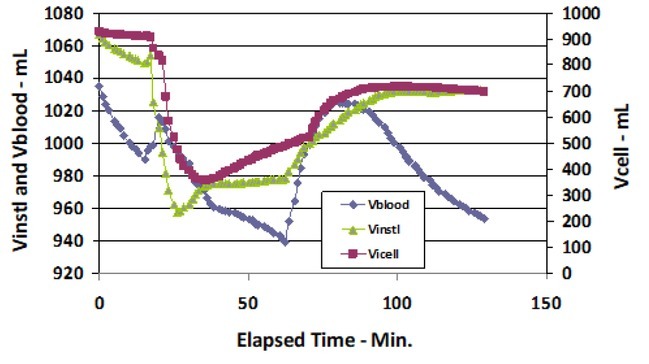
Vblood, Vinst and Vcell compartment volumes (mL) of the calf during the total DTM treatment and recovery periods vs. Elapsed Time (min).

The Ri, Re and Cm values are used to calculate the separate intravascular, interstitial and intracellular fluid compartment volumes of the calf, as given in Table 1 and Figures 4 and 5, using a custom program [13]. Vblood is the intravascular fluid compartment of the calf. Vcell and Vinstl are the intracellular and interstitial compartments, respectively. All volumes and changes in volume are in mL.

Table 1 provides the measured Vblood, Vcell and Vinstl (all in mL) at the start and end of each phase of the DTM therapy. Table 1 also shows the amount of fluid increase or decrease (mL) during each phase of DTM. All three fluid compartments lose fluid during the 15-minute control/ instrumentation period when the leg is placed on a chair. At the time when the DTM is terminated Vcell and Vinstl have both lost volume -91 and - 60 mL, respectively, while Vblood has increased by 15 mL.

During the time period Elapsed Time 60 to 120 minutes, when the subject sat upright, Vcell increased by 146 mL and Vinstl gained 31 mL. During this period Vblood decreased an additional 49 mL.

Figure 4 is a plot of one time period (Elapsed Time 0 - 40 min.) to better illustrate the dynamic response of the calf fluid compartments to DTM. DTM was found to be effective even though it was only applied for 5 min. This observation is consistent with other massage therapies that have been applied for short periods of time. Zhong et al. [14] tabulates several studies with ~5 min. application times that significantly improve the physiological, neurological and psychological feelings of the patients.

There is a time delay between the time of starting DTM and the time at which the external massage force affects a given compartment volume. Vblood loses fluid immediately upon the start of DTM. The effect of DTM is delayed approximately 1 min for Vinstl and 2 min for Vcell, respectively.

Figure 5 shows the three compartment volumes, illustrated in Figure 4, during the total duration of the DTM treatment period. This Figure 5 illustrates the volume changes after cessation of DTM, the placement of the leg in an upright position and during the extended recovery period.

## 2 The effect of DTM, removing fluid from a given compartment, continues for a prolonged period of time after termination of DTM

Between Elapsed Times 20 min. and 60 min., the leg remained horizontal. All three compartments lost fluid for 10 min. At Elapsed Time 30 min. both Vinstl and Vcell increased in volume. Vblood continued to lose volume until the leg was repositioned on the floor.

The prolonged recovery period found in this study is similar to that reported by Roffay et al. [15]. following changes in cell osmosis. They found that it may take up to 2 hours for the cell membrane tension to fully recover and return to its equilibrium state.

### Mechanisms of DTM

Several authors [15,16,17] have set forth possible mechanisms of massage that may explain the above responses to DTM. A brief summary of these mechanisms is given below to provide additional insight regarding the application of DTM.

### Equilibrium or "healthy" state of cells

Downey [15] describes "healthy" cells as being spherical in shape with a somewhat rough membrane when at their equilibrium state. The cell membrane is partially folded and partially unfolded at equilibrium. The cell membrane contains numerous transmembrane channels which become "active" or "inactive" in transporting electrolytes into or out of the cell to maintain an osmotic equilibrium across the cell membrane.

### Effect of externally applied force upon cells

Hunt et.al. [16] suggests that when an external force is applied to the cell, it may force the cells to change shape and reduce or increase the extent of cell membrane folding. This causes the cells to increase or decrease the number of cell membrane channels that pump ions and electrolytes into or out of the cell in order to reestablish the cell's osmotic equilibrium state.

### Prolonged recovery period after cessation of externally applied force

Roffay et. al. [17] reports that the effects of osmotic changes caused by an external force to the cells change the cell's volume which may take a prolonged period of time to dissipate. They found that cell volume slowly started recovery after about 15 min. but may take as much as 2 hours to be complete.

## Conclusions

This pilot study has demonstrated that DTM does affect all three fluid compartments of the calf which can be quantified using bioimpedance spectroscopy.

It quantifies the amount of fluids present in and transferred between each compartment before, during and after DTM.

Possible mechanisms of how the cell structure is changed by DTM and how it may alter the intracellular, interstitial and intravascular compartment volumes are summarized from the literature.

This information may be useful in the study of massage and other fluid management techniques. Additional research should be done using the same EIS systems to confirm these results and their applications to other clinical and environmental conditions.
